# Refinement of machine learning arterial waveform models for predicting blood loss in canines

**DOI:** 10.3389/frai.2024.1408029

**Published:** 2024-08-21

**Authors:** Jose M. Gonzalez, Thomas H. Edwards, Guillaume L. Hoareau, Eric J. Snider

**Affiliations:** ^1^U.S. Army Institute of Surgical Research, JBSA Fort Sam Houston, San Antonio, TX, United States; ^2^School of Veterinary Medicine, Texas A&M University, College Station, TX, United States; ^3^Department of Biomedical Engineering, University of Utah Health, Salt Lake City, UT, United States; ^4^Nora Eccles-Harrison Cardiovascular Research and Training Institute, University of Utah Health, Salt Lake City, UT, United States; ^5^Department of Emergency Medicine, University of Utah Health, Salt Lake City, UT, United States

**Keywords:** hemorrhage, advanced monitoring, machine learning, feature extraction, canines, military medicine, shock, predictive modeling

## Abstract

**Introduction:**

Hemorrhage remains a leading cause of death in civilian and military trauma. Hemorrhages also extend to military working dogs, who can experience injuries similar to those of the humans they work alongside. Unfortunately, current physiological monitoring is often inadequate for early detection of hemorrhage. Here, we evaluate if features extracted from the arterial waveform can allow for early hemorrhage prediction and improved intervention in canines.

**Methods:**

In this effort, we extracted more than 1,900 features from an arterial waveform in canine hemorrhage datasets prior to hemorrhage, during hemorrhage, and during a shock hold period. Different features were used as input to decision tree machine learning (ML) model architectures to track three model predictors—total blood loss volume, estimated percent blood loss, and area under the time versus hemorrhaged blood volume curve.

**Results:**

ML models were successfully developed for total and estimated percent blood loss, with the total blood loss having a higher correlation coefficient. The area predictors were unsuccessful at being directly predicted by decision tree ML models but could be calculated indirectly from the ML prediction models for blood loss. Overall, the area under the hemorrhage curve had the highest sensitivity for detecting hemorrhage at approximately 4 min after hemorrhage onset, compared to more than 45 min before detection based on mean arterial pressure.

**Conclusion:**

ML methods successfully tracked hemorrhage and provided earlier prediction in canines, potentially improving hemorrhage detection and objectifying triage for veterinary medicine. Further, its use can potentially be extended to human use with proper training datasets.

## Introduction

Physiological sensors being used to monitor vital signs during hemorrhagic shock are critical for patient care. Compensatory mechanisms of an individual mask a patient’s actual clinical status, often resulting in inadequate or delayed treatments, exacerbated by remote settings frequently faced in military medicine. Blood pressure is a commonly used clinical measurement to assess a patient’s hemodynamic status, but it fails to respond quickly to ongoing hemorrhage as well as fails to provide a status of tissue oxygenation due to loss of blood (Convertino et al., 2021). Predicting an individual’s oncoming decompensation due to hemorrhage continues to be a challenge. There is still an unmet medical need for the development of prediction metrics that can detect hemorrhage early for better resuscitation, as well as assess hemorrhaged volume-duration magnitude.

Due to limitations on data generation to develop hemorrhage prediction metrics for humans, studies will often be done in animals. The focus of this study was canines experiencing controlled hemorrhages. Datasets obtained in canines can provide proof that the methodology of extracting features from the arterial waveform for creating prediction metrics for an earlier indication of oncoming decompensation is feasible. This will provide a roadmap for applying similar processes on other animal datasets such as swine which share related anatomic and physiologic characteristics to humans (Swindle et al., 2012). In addition, military working dogs (MWDs) in combat zones are susceptible to similar injuries as service members, such as ballistics, blunt trauma, and explosive injuries (Baker et al., 2009). Human health care providers (HCP) are often the only medical assets near a MWD at the point of injury. Initial care at the point of injury is usually rendered by HCPs and continues to be provided through different levels of care until the MWD can reach veterinary providers (Giles, 2016; Edwards et al., 2021). Therefore, developing a metric to further assist non-specialized HCPs and veterinary teams in determining when a canine may be losing blood will increase the treatment quality and decrease MWD mortality.

Toward this, classical ML paired with feature extraction approaches can provide an explainable methodology for developing physiologic prediction models. ML models such as this require a feed of available physiological data and can operate at a low-cost computation setup compared to larger deep-learning neural network model types (Jordan and Mitchell, 2015). ML model explainability and computational efficiency are helped by using feature extraction, which uses a variety of calculations to measure durations, magnitude difference, variability, etc., between waveform features to reduce high frequency, computational intensive waveforms to only a few features per wavelength (Guyon and Elisseeff, 2006). When paired with ML models, these extracted features have been previously used as inputs predictors for calculating a desired physiological metric response (Hatib et al., 2018). We hypothesize that robust features can be extracted from canine arterial blood pressure datasets and used to calculate prediction metrics that can predict oncoming decompensation prior to the blood pressure metric, the current gold standard.

## Materials and methods

### Canine hemorrhage study

This study was approved by the Institutional Animal Care and Use Committee at the University of Utah (21–01012) and received second level approval from the Department of Defense. Six adult, purpose-bred male canines aged 1–3 years were acclimated in a laboratory animal facility for at least 1 month prior to the start of the experiment. The dogs were premedicated with midazolam (0.3 mg/kg IV) and anesthetized with a combination of fentanyl (5 mcg/kg IV) and propofol (2–4 mg/kg IV). Dogs were maintained on total intravenous anesthesia consisting of propofol (1–20 mg/kg/h), midazolam (0.1–0.5 mg/kg/h), and fentanyl (0.05–0.3 mcg/kg/min). An orotracheal tube was placed and animals were ventilated, as needed, to maintain a pulse oximetry reading of 95–99% and an end-tidal carbon dioxide reading of 35–45 mmHg. A triple lumen catheter was placed in the jugular vein and a 5 French catheter was inserted under ultrasound guidance into the femoral artery.

To induce hemorrhagic shock, venous blood was removed through the jugular catheter over 1 h until either a mean arterial pressure of 35–50 mmHg or 40% of the estimated total blood volume was reached, whichever occurred first. The dogs were maintained in a state of hemorrhagic shock for 45 min and then given a randomized resuscitation fluid strategy. After recovery from hemorrhagic shock and resuscitation, all canines were allowed at least 4 weeks to fully recuperate between studies, allowing for multiple technical replicates for each canine study (6 canines, each undergoing 5 rounds of hemorrhaging and resuscitation). The canines were adopted upon the completion of the study. A full description of the study protocol has recently been published (Ford et al., 2023). The data were captured using a data acquisition device at 1 kHz (PowerLabs, ADInstruments, Sydney, Australia). Notable timepoints such as baseline, start of hemorrhage, end of hemorrhage, and end of shock hold were marked and used for identifying study phases. The resuscitation portion of the canine data was not used for this ML development study. The data were downsampled to 500 Hz and then exported as text files for the following feature extraction process.

### Feature extraction and predictor selection

Arterial waveform datasets of canine subjects were used to extract a multitude of different features using MATLAB (v2023a, MathWorks, Natick, MA, United States). Each arterial waveform was filtered using a finite impulse response (FIR) window lowpass filter. The pulse foot, systolic peak, half-rise between the pulse foot and systolic peak, first inflection point, and the end point of the waveform segment (pulse foot of the following waveform segment) were identified for each waveform segment of the arterial waveform (Bedolla et al., 2023). If a waveform segment lacked an inflection point, the half-drop between the systolic peak and the following pulse foot was calculated. Using these extracted waveform landmarks, 1901 features were calculated from each waveform of each canine subject based on previous research efforts (Hatib et al., 2018; Gupta et al., 2022) as well as features developed internally. A detailed explanation of the various feature types is described in Supplementary material.

Various hemorrhagic metrics were evaluated to be predicted by the extracted features. A blood loss volume metric (BLVM) was developed for predicting impending hemorrhage on a 0–1 scale. This prediction was calculated as 1 minus the hemorrhage volume at a given time, t, over the total hemorrhage volume of the study. This was calculated across the baseline and hemorrhage region using Equation 1. The hold region of the study defaulted to BLVM being 0, as maximal hemorrhage volume was maintained with no active hemorrhage.


(1) $$BLVM=1-\frac{HemorrhagedVolume\left(t\right)}{TotalShedHemorrhagedVolume}$$

The total blood loss and the canines’ weight were available in the dataset, so a prediction of percent estimated blood loss (PEBL) was developed. The total blood volume of the dog used to calculate PEBL was estimated as 80 mL/kg (Vos et al., 2016). PEBL was calculated as the total hemorrhage volume at time t, divided by the total estimated blood volume of the canine subject. PEBL is shown in Equation 2.


(2) $$PEBL=\frac{\mathrm{Hemorrhaged Volume}\;\left(t\right)}{80\;\frac{mL}{kg}\;x\;canineweight\;\left(kg\right)}$$

**Figure 1:**
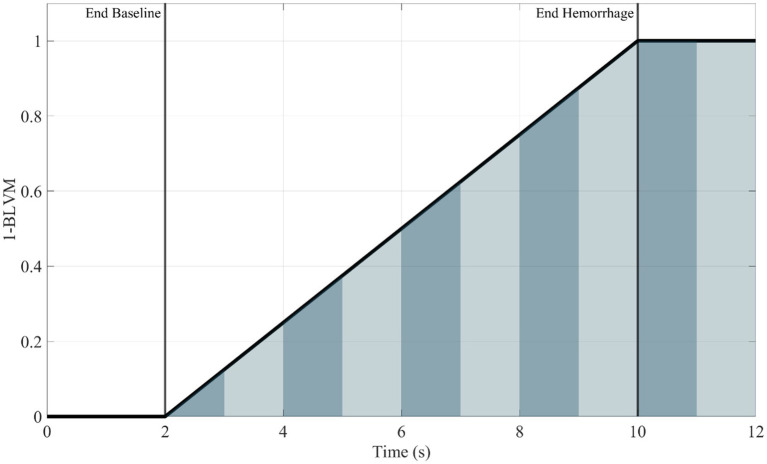
Representative plot (1—BLVM vs. time) demonstrating integral slices under the curve. The area under the curve for the HemArea prediction was calculated using small changes in time as an integral slice. A summation of these slices was plotted over time, and the ML model was used to predict this accumulation of area under the curve over time. Area slices are alternated in color for visualization purposes to distinguish between each slice.

The final metric was developed to track the accumulation of hemorrhage over time. Two distinct methods were developed for integral-based hemorrhage area predictions. The first being the area under the hemorrhage volume (y-axis = hemorrhage volume and x-axis = duration) region (HemArea) as this would track the overall hemorrhage burden into the hold region as the volume hemorrhaged would stay constant. Still, the integral would increase as the canine hemorrhaged volume-duration increased over a period of time at that distinct volume. A ML model was developed to predict the accumulating HemArea over time directly. A separate approach was taken to calculate HemArea directly using BLVM model predictions, as this metric calculates the hemorrhage volume. The BLVM-based method used linear regression of the prediction outputs as BLVM is based on a 0 to 1 scale. BLVM is subtracted by 1 and multiplied by time to calculate an integral slice, then summed to calculate the total accumulating area. The HemArea calculation is shown in Equation 3 and diagrammed in Figure 1.


(3) HemArea=∑|BLVM−1|×Δt

All predictions calculated in this study were smoothed using a moving mean average window size of 500 data points to generalize predictions trends. The predictions were assessed by standard linear regression vs. ground truth calculations, and any prediction that had significant outliers went through a linear regression with robust options to reduce sensitivity to outliers compared to standard linear regression.

### Machine learning tuning

The ML models used were decided by previous work (Bedolla et al., 2023) in which a bagged tree ML model performed best and was used as the basis for developing all ML models in this study. The ML models were first developed with all the canine data to create generalized canine ML models. The features were ranked using the minimal-redundancy-maximum-relevancy (MRMR) ranking criterion in the MATLAB (v2023a, MathWorks, Natick, MA, United States) Regression Learner Toolbox. Once the features and the models were trained, they were tested using a cross-validation technique known as leave one subject out (LOSO) to account for bias in the testing. The LOSO process was done six times, leaving one blind test subject out of the training, and then the blind subject was used as testing data on this trained ML model. Each model was initially run with the top 10 features and either halved or doubled based on their performance. This process was repeated twice for three different ML models developed for each hemorrhage prediction metric. The maximum number of features used as an input to the ML model was decided when the R-squared values started to plateau or drop from the previous run of the same ML model, with only the number of features being used changing up to a maximum of three different setups. Each ML model was held constant with 1 learner. The leaf sizes used to train the bagged tree ML models were 4, 8, 12, and 16. Each prediction metric resulted in a total of 12 trained ML models (3 different number of features × 4 leaf sizes). Each ML prediction metric model was blind-tested across 6 LOSOs with five replicates for a single-blind subject. This resulted in a total of 30 blind datasets (6 LOSOs × 5 subject replicates) used for validation across the 12 ML cross-fold validation models for each prediction metric.

### Machine learning performance metrics

After performing the LOSO test for each trained ML model for each respective prediction, the R-squared and the root mean squared error (RMSE) were measured to determine the best-tuned ML model for each respective hemorrhagic prediction metric. An R-squared and RMSE value were gathered for each respective round and averaged across each respective canine. To be able to directly compare RMSE values of the best performing ML models developed for the various prediction metrics, a normalized RMSE was also calculated. The normalized RMSE was calculated by dividing the raw RMSE value for each dataset by the range of raw data experienced by the prediction metric of interest. This normalization calculation is shown in Equation 4.


(4) $$RMSE_{Normalized}=\frac{{\mathrm{RMSE}}_{Data}}{Data_{\max}-Data_{\min}}$$

In addition, receiver operating characteristic (ROC) curves and the area under the ROC curve (AUROC) were calculated for the best model configurations for each ML model output based on their ability to distinguish baseline and hemorrhage states in the datasets accurately. Different determination thresholds were used to construct these curves. A similar methodology was then used to estimate how early hemorrhage was detected for each ML model predictor. Briefly, datasets were normalized to 100 data points for each of the baseline, hemorrhage, and hemorrhagic shock hold region for each canine dataset. Each data point was categorized as positive or negative for hemorrhage based on a threshold value set at the 25th percentile of baseline values for each hemorrhage dataset. For metrics that increased in value as opposed to dropping in value during hemorrhage, the 75th percentile of baseline values was used. Hemorrhage detection was determined by five consecutive predictions of hemorrhage for each ML metric, as well as for MAP.

## Results

### Optimization of machine learning models for each predictor

**Figure 2:**
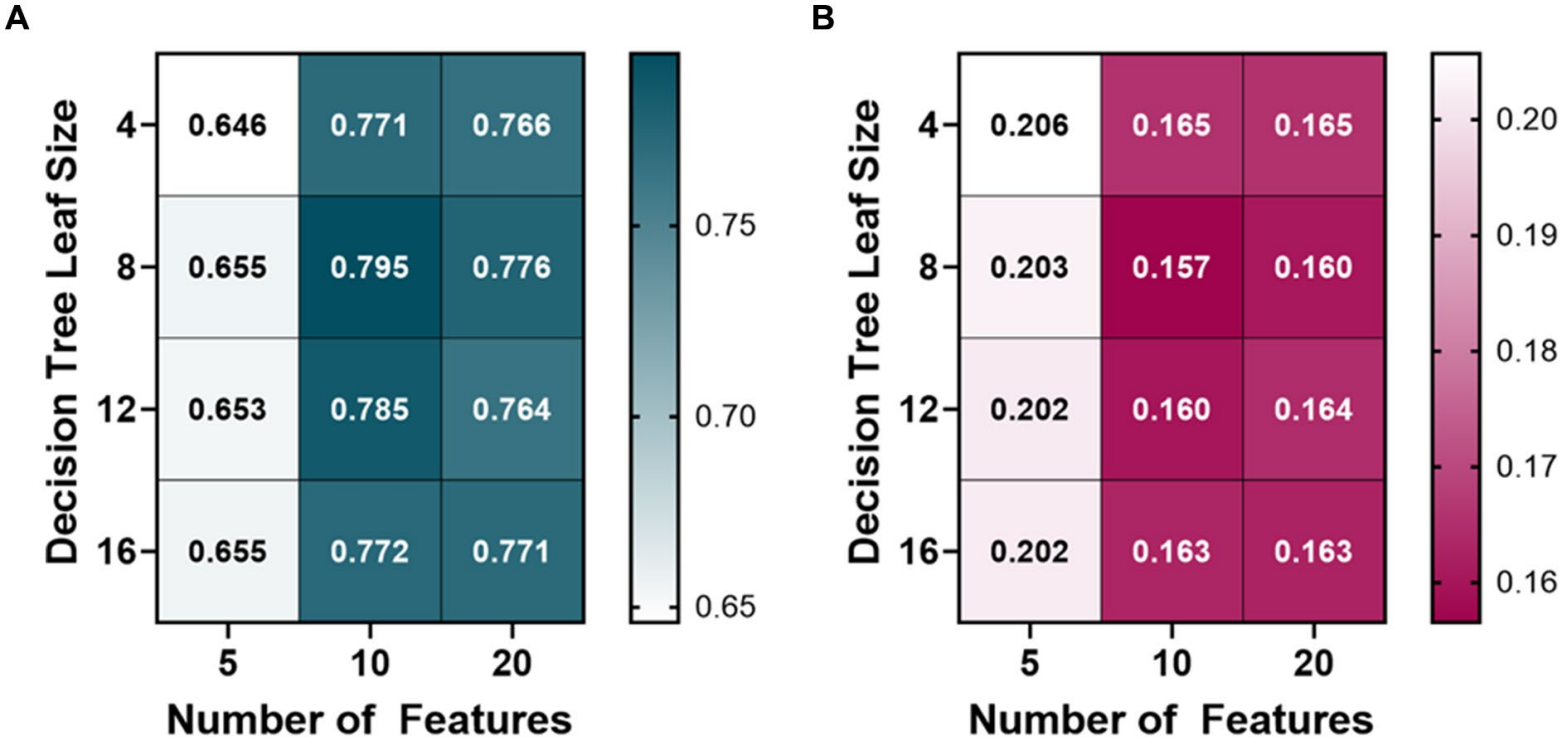
Model optimization results for predicting a blood loss volume metric (BLVM). BLVM ranges from 1 (no blood loss) to 0 (maximum blood loss). Decision tree models were trained with 4, 8, 12, and 16 minimum leaf sizes while using 5, 10, or 20 extracted arterial waveform features as model input. Average results are shown for each model as **(A)** R-squared and **(B)** root mean squared error values.

A variety of bagged tree ML models were trained and compared for predicting each metric. The BLVM metric was initially trained using 10 features, with a decision tree leaf size between 4 and 16. The number of features was then doubled, and the R-squared values were compared to the 10 feature ML models (Figure 2). Overall, the 10 feature ML models performed better than the 20 feature ML models (Average R-squared value of 0.783 vs. 0.77), so the number of features was capped at 20. The ML model’s features were then halved to see if a similar performance could be had with fewer features. The BLVM ML models with 5 features performed worse than the 10-feature ML models. Thus, the best-performing ML model for BLVM was with 10 features and a minimum leaf size of 8, reaching an R-squared value of approximately 0.795 with the lowest RMSE at 0.157.

**Figure 3:**
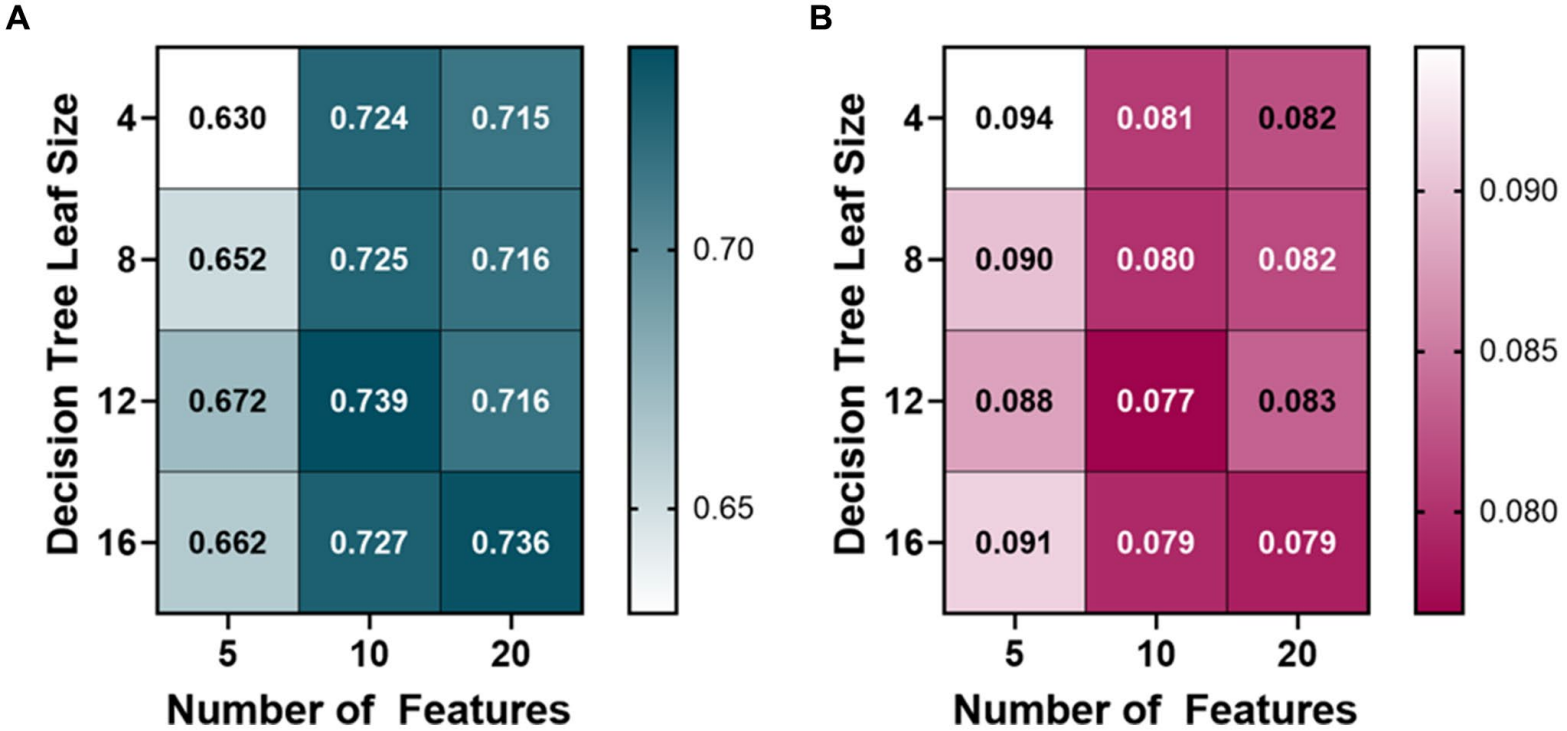
Model optimization results for predicting percent estimated blood loss (PEBL). PEBL ranges from 0 (no blood loss) to 1 (total estimated blood loss). Decision tree models were trained with 4, 8, 12, and 16 minimum leaf sizes while using 5, 10, or 20 extracted arterial waveform features as a model input. Average results are shown for each model as **(A)** R-squared and **(B)** root mean squared error values.

The PEBL bagged tree ML models also began at 10 features and were trained with a leaf size of 4, 8, 12, and 16 (Figure 3). The average R-squared value of the 10 feature ML models was 0.728, so the features were then doubled to see how the performance would change. The 20 feature ML models had an average R-squared value of 0.723, decreasing over the 10 feature ML models. The number of features was then halved from 10 to 5 to see if the PEBL ML models could perform similarly with fewer features. The 5 feature ML models performed worse with an average R-squared value of 0.653. The best-performing ML model for predicting PEBL was at 10 features, 12 leaf size at 0.739 R-squared value, followed closely by 20 features, 16 leaf size with both ML models having an R-squared value of 0.736.

**Figure 4:**
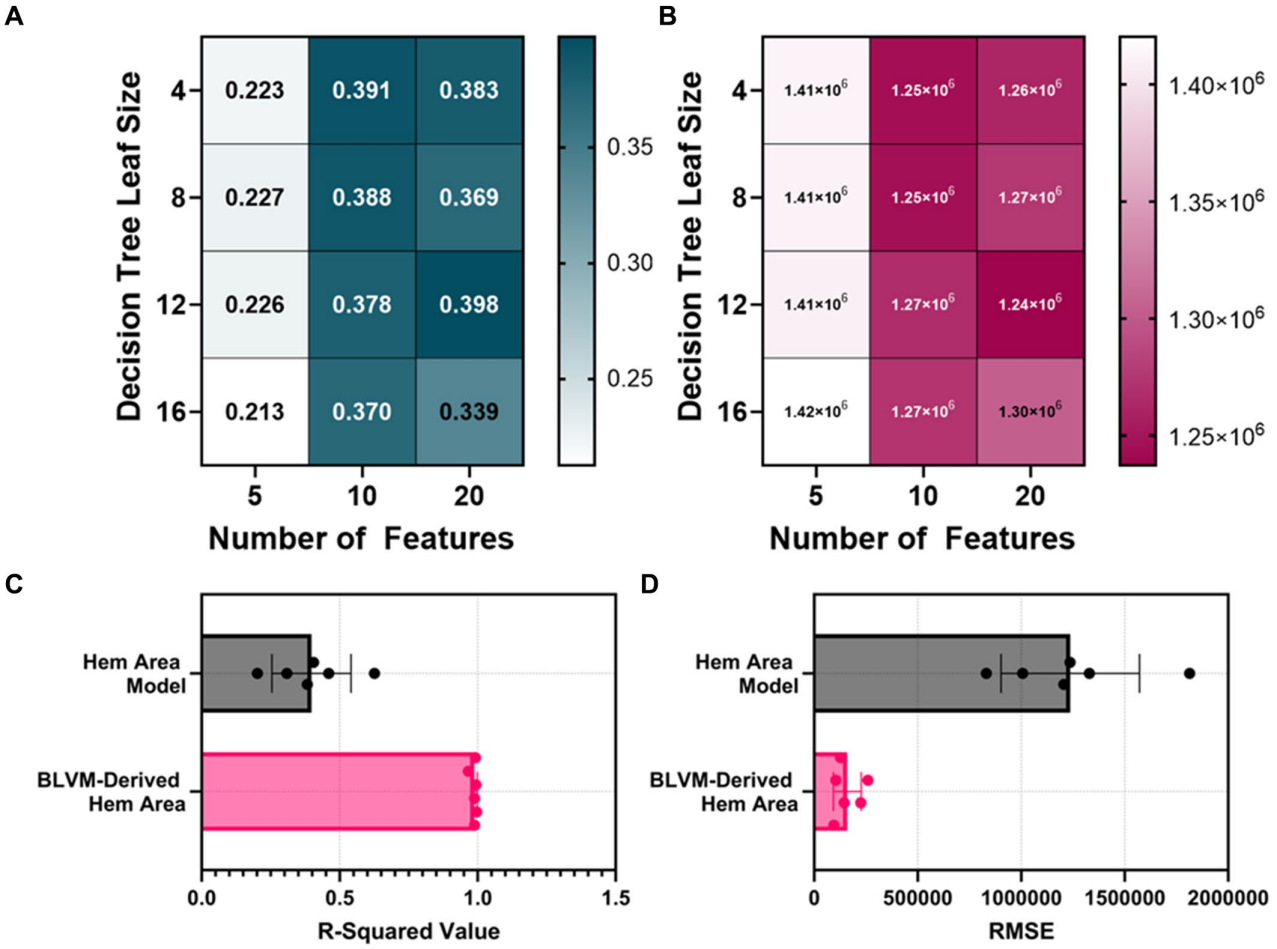
Model optimization results for predicting hemorrhage area (HemArea). Hem Area is defined as the area between no blood loss and current blood loss across the time axis. This results in HemArea being a factor of hemorrhage magnitude and duration and increases in value as either hemorrhage volume or hemorrhage time increases. **(A,B)** Decision tree models were trained with 4, 8, 12, and 16 minimum leaf sizes while using 5, 10, or 20 extracted arterial waveform features as a model input for predicting HemArea. Results are shown for **(A)** R-squared and **(B)** RMSE. **(C,D)** The best performing HemArea ML model were compared to BLVM-Derived HemArea calculations for **(C)** R-squared and **(D)** RMSE values. Results are shown as average with error bars denoting standard deviation.

The HemArea ML models took two different approaches. The first being the direct prediction of the HemArea using a bagged tree ML model. The ML models were initially trained with 10 features and had an average R-squared value of 0.3825 between the four models (4, 8, 12, 16 leaf size, Figures 4A,B). The number of features used to train the models was then doubled, and the average R-squared value of the 20 feature HemArea ML models was 0.3725. As that value was a drop over the 10 feature ML models, the cap was set at 20 features. Five feature ML models were then tested and had an average R-squared value of 0.2225. Due to the poor overall performance, we compared calculation of HemArea by measuring the area under the BLVM vs. time plot. This was performed using the optimal BLVM prediction model parameters of 10 input features and a leaf size of 8. The R-squared value was much higher at 0.988 compared to 0.398 R-squared value for the best performing HemArea ML model (20 input features, 12 leaf size, Figure 4C). RMSE for the BLVM-derived HemArea was reduced to 13% of the HemArea ML model (Figure 4D).

### Comparison of model predictors

Optimal ML model parameters were selected for each predictor type based on R-squared values. The BLVM model with the best performance used 10 features and a leaf size of 8. The best-performing PEBL model used 20 features with a leaf size of 12. The HemArea model directly predicting the area under the curve as it increased had the best performance using 10 features and a leaf size of 4, but its performance remained low and was not considered further. The BLVM-derived HemArea was calculated with the optimal BLVM model (10 features and a leaf size of 8). The performance metrics of R-squared and RMSE values for each model are shown in Table 1. Overall, the BLVM model outperformed PEBL (0.795 vs. 0.739), but both fell far short compared to the BLVM-Derived HemArea metric (0.988) based on R-squared scores (Table 1). RMSE values were normalized to each metrics value range so that they could be compared across models. Overall, BLVM-derived HemArea has the strongest Normalized RMSE at 0.00326 while all the other models faired similarly, with values ranging from 0.162 to 0.197 (Table 1).

**Table 1:** Summary of model performance metrics for each trained model type.

	R-Squared	RMSE	Normalized RMSE
BLVM	0.795	0.157	0.162
PEBL	0.739	0.0768	0.176
BLVM-derived HemArea	0.988	159,485	0.00326

R-squared and RMSE values for each top performing model averaged across the LOSO test results are shown.

**Figure 5:**
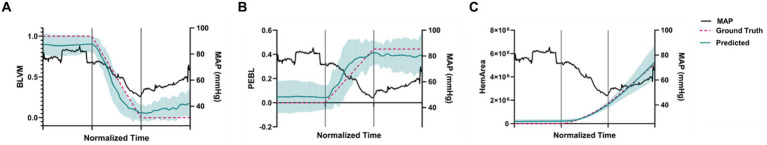
Predictions across the animal model experimental phases for each model. The time axis is normalized so that the first third of the plot is baseline results, the second third is during hemorrhage, and the final third is the shock hold region. Teal values indicate predictions and pink values represent ground truth across all subjects for **(A)** BLVM, **(B)** PEBL, and **(C)** BLVM-derived HemArea. (*n* = 5 technical replicates, *n* = 6 subjects). Error bars denote the standard deviation of time-aligned data for the predictions. Mean arterial pressure is shown on the secondary axis in black for comparison.

The average of all canines and their different prediction metrics across the experimental phases are shown in Figure 5. All the prediction metrics were normalized into three windows due to varying lengths of data recordings. These regions were split into (i) baseline, (ii) hemorrhage, and (iii) the shock hold region. The prediction metrics are plotted alongside the MAP for comparison. The BLVM tracked the ground truth and began dropping prior to the MAP during the hemorrhage region. PEBL also tracked the ground truth and began to increase before the MAP decreases during the hemorrhage region. The HemArea prediction began to rise prior to the MAP dropping. The HemArea metric also had a unique behavior in the hemorrhage region and into the shock hold region. During baseline, the standard deviations were close to the predicted values, while during hemorrhage and into the shock hold region, the standard deviations increased noticeably. This was unique to the HemArea predictions and was not seen in BLVM and PEBL.

**Figure 6:**
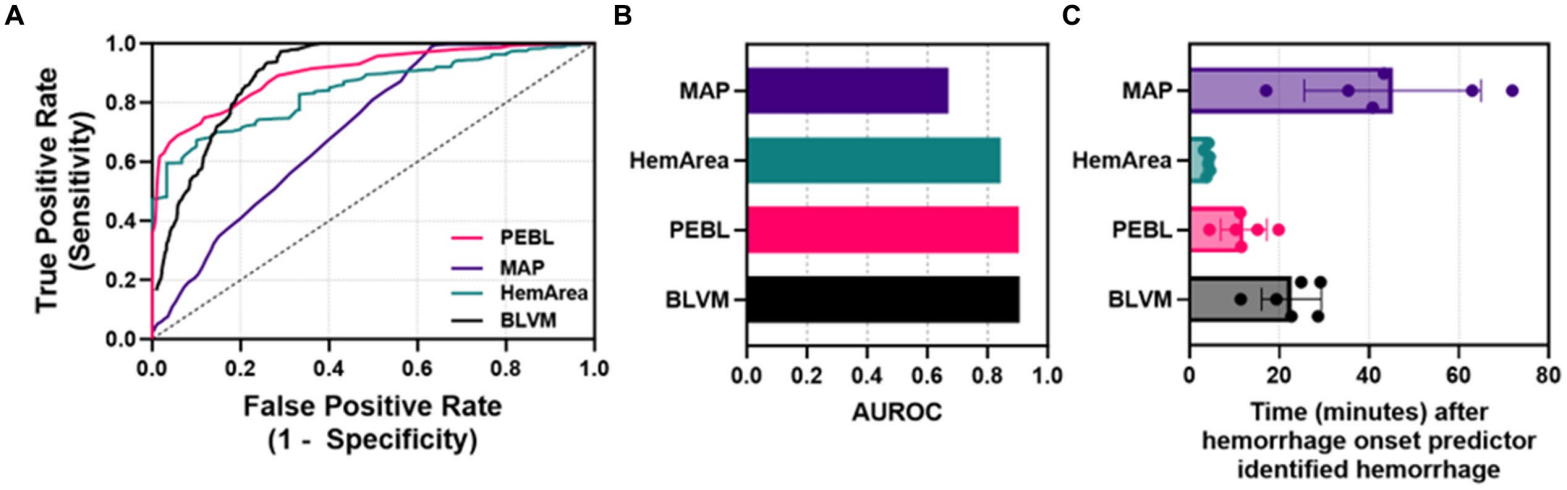
Characterization of each predictor for detecting hemorrhage. **(A)** ROC curves for each ML predictor and MAP. **(B)** Areas under the ROC curve were quantified for each ML predictor and MAP. **(C)** Quantified time for detecting hemorrhage for MAP, HemArea, PEBL, and BLVM, based on consistent categorical hemorrhage classification.

Next, ROC analyses were performed to determine how well each metric could distinguish baseline and hemorrhage categorical outcomes (Figure 6A). Overall, BLVM and PEBL had similar performance, with each reaching approximately with 0.9 AUROC scores (Figure 6B). HemArea has a slightly worse performance, with AUROC reaching approximately 0.84. The lowest performing predictor was MAP, with AUROC only at 0.67. Detection of consistent hemorrhage identification was predicted based on five consecutive indications of hemorrhage by each ML model. MAP as a predictor required more than 45 min after hemorrhage onset for consistent detection on average (Figure 6C). BLVM improved detection performance by identifying hemorrhage after only 23 min. The other two metrics, PEBL and HemArea, were much improved at hemorrhage detection, with 12.0 and 4.0 min after hemorrhage onset. While HemArea may have had the quickest response, all the prediction metrics responded to hemorrhage quicker than the MAP.

## Discussion

Since hemorrhage remains a leading cause of mortality after trauma, means for earlier injury detection and hemorrhage quantification could be life-saving for future civilian, veterinary, and military medicine. Many methods have been proposed for early hemorrhage detection, such as compensatory reserve measurement (CRM), an advanced monitoring algorithm for tracking compensatory status prior to shock (Convertino and Schiller, 2017). However, this algorithm was not developed for tracking compensatory status for canines undergoing hemorrhage. As a result, we developed a blood loss metric to identify an ongoing hemorrhage in a canine subject. Here, we tuned BLVM as well as introduced and evaluated other metrics that will offer enhanced advanced monitoring strategies that ideally would apply to humans and other species for universal use. In this effort, we detailed a range of prediction and ML strategies and determined if any were suitable for measuring hemorrhaged volume-duration magnitude and earlier hemorrhage prediction especially in trauma use case where hemorrhage status is often not known.

We previously evaluated classical ML strategies for successfully tracking the CRM in humans and found that a decision tree model was most suitable for this application (Bedolla et al., 2023). Unfortunately, a ML model could not be evaluated for measuring compensatory status as the canines were anesthetized during hemorrhage, so determining when the animal reached decompensation was unknown, unlike the human lower body negative pressure chamber datasets used to develop CRM where patient decompensation status was known as the patient was conscious and able to communicate precursor symptoms of decompensation. Instead, the three metrics we evaluated here were focused on hemorrhage quantification. BLVM and PEBL mirror the equations used for CRM, with each ranging from 0 to 1 or 100% based on the extent of maximum hemorrhage or fraction of estimated blood volume, respectively. ML models were successfully developed and optimized for each approach. Additionally, the decision tree configuration over the range of setups we evaluated only minimally altered model performance.

Conversely, we considered an additional metric based on the integral of the hemorrhage volume vs. time region. This additional metric was considered to incorporate time into a quantification of the hemorrhage, which is not included in metrics such as BLVM and PEBL. This metric accounts for duration of shock, which may affect the response of the ML model’s as well as the ML model’s earlier prediction time. This metric was unique from the original CRM approach as this metric was not capped between 0 and 1. Instead, this potential integral-based metric could allow for better patient triage as subjects in a hypovolemic state for 5 min will score much lower than someone in this state for an hour, which would not be distinguished by BLVM or PEBL. However, additional research will be needed to show how a duration and magnitude triage metric such as this would trend with clinical trauma cases. ML models for this approach did not result in strong correlations, and we instead calculated hemorrhage area from BLVM. This approach resulted in strong prediction, but more robust model formats or deep learning approaches may allow for directly predicting the area metric. With models for each output prediction, we evaluated which models were optimal for early hemorrhage prediction. BLVM allowed for approximately 50% quicker hemorrhage prediction compared to MAP. PEBL and HemArea further improved prediction time at approximately 75% quicker and 90%, respectively. HemArea has the widest operating range of values and potentially has the highest sensitivity to changes, resulting in the earliest prediction time.

For BLVM, it is relative to the maximum hemorrhage used in this study, which is an arbitrary point when translating these metrics beyond this study. Instead, PEBL allows for a number relative to estimated blood volume based on animal weight, which more easily translates outside this study. However, PEBL and BLVM suffer in terms of triage as they do not provide a means of distinguishing casualties with regard to time in hypovolemia. As the HemArea metric can be derived from BLVM, both predictors could be used in unison: PEBL or BLVM for quick hemorrhage detection and HemArea for distinguishing hemorrhaged volume-duration magnitude when needing to triage injury levels when resources are limited, such as in combat casualty care.

There are some limitations with the current study design that can be improved. The canines in this study were anesthetized and only had a single trauma/injury. The addition of more injuries or different anesthetic approaches can alter the underlying physiology and likely the outcomes of the ML models. Thus, additional data is required to create more generalized ML models for a more robust trauma use case. The canine datasets are also limited in size and scope. We performed LOSO cross-validation approaches to extend the data as best possible. Still, only 6 subjects of data may not result in robust enough models for real-time implementation at this time. Further, all animals were undergoing a controlled hemorrhage to a set percent volume or target MAP, so additional data is needed to expand the model training to include different injury severities and types, such as uncontrolled hemorrhage. Second, the methodology currently relies on an arterial waveform for making ML predictions. Arterial lines are not placed early in many trauma patients, so the availability of an arterial line will limit the utility of these metrics for triage and hemorrhage prediction. Future endeavors will expand this work toward non-invasive waveforms that could be used as inputs for the ML models. Third, the dicrotic notch is a key landmark used during feature extraction methods, but it sometimes cannot be identified resulting in estimating its location currently. More work is needed to determine how that estimate should be considered – mid-point between the systole and diastole or skewed more in either direction. This can be used as a hyperparamter during optimization studies to more effectively identify its placement. Lastly, these metrics have only been evaluated for this canine use-case. Future work will assess if these metrics can be developed for use in swine. Additional work will also look at how features used to feed the ML model’s correlate to physiological processes in the vascular system to improve on the clinical basis for the ML models.

## Conclusion

ML algorithm models can be developed and tuned to accurately predict blood loss in canine. Models for BLVM, PEBL, and HemArea after optimization across the number of input features and leaf sizes resulted in high R-squared values and low RMSE. These models can assist healthcare providers in MWD treatments at point-of-injury locations and may improve MWDs’ successful resuscitation rates. The ML feature extraction and model development success in the canine model can pave the way to similar developments in swine models, which are physiologically and anatomically similar to humans. This is a critical next step in model clinical translation.

## Data availability statement

The datasets presented in this article are not readily available because they have been collected and maintained in a government-controlled database that is located at the US Army Institute of Surgical Research. As such, these data can be made available through the development of a Cooperative Research & Development Agreement (CRADA) with the corresponding author. Requests to access the datasets should be directed to ES, eric.j.snider3.civ@health.mil.

## Ethics statement

The animal study was approved by the Institutional Animal Care and Use Committee at the University of Utah. The study was conducted in accordance with the local legislation and institutional requirements.

## Author contributions

JG: Conceptualization, Data curation, Formal analysis, Methodology, Writing – original draft, Writing – review & editing. TE: Funding acquisition, Writing – original draft, Writing – review & editing. GH: Funding acquisition, Writing – original draft, Writing – review & editing. ES: Conceptualization, Funding acquisition, Investigation, Methodology, Resources, Supervision, Visualization, Writing – original draft, Writing – review & editing.
