# Supplementary material for: Refinement of machine learning arterial waveform models for predicting blood loss in canines
Source: Front Artif Intell. 2024 Aug 21;7:1408029. doi: 10.3389/frai.2024.1408029 (PMC11371769; doi:10.3389/frai.2024.1408029)
Supplement: Supplementary file 1 [file Table1.DOCX]

**Supplementary Information**

Table S1. Summary of general categories of features with descriptions and examples of each feature category. Feature calculations were based on publications by Kurylyak et al. [1], Gupta et al. [2], Cannesson et al., as well as feature developed internally at the United States Army Institute of Surgical Research [3]. For additional interest in the feature set, contact the corresponding author of the study.

| **Feature Category** | **Description** | **Examples*** | **Quantity** |
| --- | --- | --- | --- |
| Base features | Feature calculated using simple mathematical manipulation of the landmarks of the BP beat | Peak to peak interval, Pulse area, average pressure of the systolic phase, ratio of the systolic to diastolic, slope at the half rise between the pulse foot and systolic peak | 42 |
| Normalization by number of samples in a window | Base features are normalized by dividing the number of samples in a window | Diastolic area divided by the number of samples in a window between the pulse foot and the end of the waveform | 11 |
| Normalization by number of samples in the entire signal | Base features are normalized by dividing the number of samples in the entire signal | Systolic decent area divided by the number of samples in the signal where the systolic decent area was calculated from | 201 |
| NODIA features | Base features are normalized by subtracting the waveform value at the inflection point | Mean of the systolic decent subtracted by the waveform value of the inflection point where the mean of the systolic decent was calculated from | 20 |
| Normalization by the width of the heartbeat pulse | Base features are normalized by dividing the width of the pulse | Pulse pressure divided by the width of the waveform where the pulse pressure was calculated from | 6 |
| Normalization by the width of the entire signal | Base features are normalized by dividing the width of the entire signal | Systolic area divided by the width of the signal where the systolic was calculated from | 6 |
| Variability (Heartbeats) | The standard deviation of the base features over a window of 20 heartbeats | Variability of the ratio between the waveform value of the systolic point and the diastolic point | 95 |
| Variability (Time) | The standard deviation of the base features over a window of 20 seconds | Variability of the average slope between the pulse foot and the systolic peak | 95 |
| Simple Delta-Change | Base features normalized by subtracting the mean value of the window from the mean of the baseline | Simple Delta-Change of the peak to peak interval | 475 |
| Complex Delta-Change (Heartbeats) | Base features normalized by subtracting the mean value of a shifting window of heartbeats from the mean of the baseline | Complex Delta-Change (20 heartbeats) of the area of the region between the pulse foot and the half rise between the pulse foot and the systolic peak | 475 |
| Complex Delta-Change (Time) | Base features normalized by subtracting the mean value of a shifting window of time from the mean of the baseline | Complex Delta-Change (20 seconds) of the systolic area | 475 |

**Supplementary References**

[1] Y. Kurylyak, F. Lamonaca, and D. Grimaldi, “A Neural Network-based method for continuous blood pressure estimation from a PPG signal,” in *2013 IEEE International instrumentation and measurement technology conference (I2MTC)*, IEEE, 2013, pp. 280–283.

[2] J. F. Gupta, S. H. Arshad, B. A. Telfer, E. J. Snider, and V. A. Convertino, “Noninvasive Monitoring of Simulated Hemorrhage and Whole Blood Resuscitation,” *Biosensors*, vol. 12, no. 12, Art. no. 12, Dec. 2022, doi: 10.3390/bios12121168.

[3] C. N. Bedolla, J. M. Gonzalez, S. J. Vega, V. A. Convertino, and E. J. Snider, “An Explainable Machine-Learning Model for Compensatory Reserve Measurement: Methods for Feature Selection and the Effects of Subject Variability,” *Bioengineering*, vol. 10, no. 5, Art. no. 5, May 2023, doi: 10.3390/bioengineering10050612.
